# Similar plasma lipidomic profile in people living with HIV treated with a darunavir-based or an integrase inhibitor-based antiretroviral therapy

**DOI:** 10.1038/s41598-019-53761-7

**Published:** 2019-11-20

**Authors:** Alvaro Mena, Elvira Clavero, José Luis Díaz-Díaz, Angeles Castro

**Affiliations:** 1 Grupo de Virología Clínica, Instituto de Investigación Biomédica de A Coruña (INIBIC)-Complejo Hospitalario Universitario de A Coruña (CHUAC), Sergas. Universidad de A Coruña (UDC), A Coruña, Spain; 2 Unidad de Enfermedades Infecciosas, Servicio de Medicina Interna, Complejo Hospitalario Universitario de A Coruña (CHUAC), A Coruña, Spain; 3 Servicio de Medicina Interna, Complejo Hospitalario Universitario de A Coruña (CHUAC), A Coruña, Spain

**Keywords:** Prognostic markers, Dyslipidaemias

## Abstract

Cardiovascular disease is an important cause of morbidity and mortality in people living with HIV (PLWH), who commonly experience lipid disturbances. The aim of this study was to determine whether the plasma lipidomic profile differs between PLWH receiving a darunavir-based ART and those receiving integrase inhibitor-based ART. This was a cross-sectional study of unselected patients for whom metabolomic analysis was performed using ultra-high-performance liquid chromatography coupled to mass spectrometry. Data for the two subgroups were compared by calculating the log2 of the fold change for each metabolite and then grouping these into the main lipid families. Sixty-two PLWH aged 49.3 ± 8.6 years (82% men) were included: 12 patients (19.4%) had hypertension, 8 (12.9%) had type 2 diabetes, 25 (41.0%) had dyslipidaemia and 9 (14.5%) were taking statins, without significant differences in all these variables between the two groups. Twenty-five (40.3%) received darunavir-based ART and 37 (59.7%) integrase inhibitor-based ART. Although the differences were not statistically significant, patients treated with darunavir-based ART had higher concentrations of total cholesterol (211 mg/dL vs 194 mg/dL), LDL-cholesterol (132 mg/dL vs 117 mg/dL) and triglycerides (155 mg/dL vs 122 mg/dL), and lower HDL-cholesterol concentration (50 mg/dL vs 52 mg/dL). The main lipid families and metabolites differed slightly between groups (log2-fold change; P-value): ceramides (−0.07; 0.49), phosphatidylinositols (−0.05; 0.63), diacylglycerols (0.10; 0.64), phosphatidylethanolamines (0.03; 0.78), triacylglycerols (0.27; 0.18) and lysophosphatidylethanolamines (0.03; 0.83). In the integrase inhibitor-based group, the use of tenofovir alafenamide fumarate significantly increases the majority of lipid fractions, when compared with tenofovir disoproxil fumarate. The lipidomic profile did not differ between PLWH treated with darunavir-based or integrase inhibitor-based ART. This was especially true for ceramides, which are involved in cardiovascular disease. Further studies are needed to study the impact of ART in lipidomic profile.

## Introduction

The incidence of cardiovascular (CV) events is increasing in people living with HIV (PLWH) for many reasons such as the improvement in life expectancy, chronic inflammatory status of the disease or toxicity of antiretroviral treatment (ART)^1^.

Dyslipidaemia is associated with increased risk of CV diseases in PLWH^2^. ART-induced dyslipidaemia is characterized by a reduction in HDL-cholesterol and an increase in LDL-cholesterol, total cholesterol and triglyceride concentrations^3,4^. These parameters are incorporated in most of the equations used to estimate CV risk, even though these scores can underestimate the risk in PLWH^5^.

Some molecular lipid species, especially ceramides, are involved in many central processes involved in the development of atherosclerosis and are significant predictors of CV death in HIV-uninfected patients with and without established CV diseases^6,7^. Ceramides have also been implicated in cell senescence and apoptosis^8^. PLWH exhibit several differences in the plasma lipid profile compared with healthy people not infected with HIV. These include increase levels of some metabolites such as diacylglycerols and reduced level of others such as sphingomyelin. However, little is known about the effects of ART on plasma lipidomic profile^9^.

Classically, protease inhibitors were associated with a worse lipid profile than are integrase inhibitors, as measured by the differences in the concentrations of cholesterol and triglycerides. However, these differences are smaller with modern protease inhibitors such as darunavir, especially when boosted with cobicistat instead of ritonavir^10^. The aim of this study was to determine whether the plasma lipidomic profiles differ between PLWH receiving a darunavir-based ART and those receiving an integrase inhibitor-based ART.

## Patients and Methods

### Ethics statement

The research protocol was conducted in accordance with the Declaration of Helsinki and the STROBE Statement^11^. It was reviewed and approved by the Regional Ethics Committee of Galicia (register code 2016/423). All participants provided written informed consent. A database was created, and all data were anonymized before analysis.

### Patients

This was a cross-sectional study of consecutive PLWH who attended a mono-centric HIV unit. In this unit, approximately 1380 PLWH with stable ART are followed. Of them, 29% received a darunavir-based regimen and 43% an integrase inhibitor-based ART. Participants were included if they were being treated with a darunavir-based or an integrase inhibitor-based ART, for at least six months, were older than 18 years old, and signed the informed consent. The exclusion criteria were: pregnant women and people with an active AIDS-defining condition or an active cancer.

Demographic data, CV risk factors and HIV characteristics (duration of HIV infection, other coinfections, CD4 + T-cell count, HIV viral load and ART) were collected. Blood samples were obtained under fasting conditions of at least 8 hours, processed and frozen at −80 °C. Participants were included in the study between January and March 2017.

### Metabolomic analysis

Serum metabolic profiles were semi-quantified using ultra-high-performance liquid chromatography coupled to mass spectrometry (UHPLC-MS) as described previously^11^. Briefly, two separate UHPLC-time of flight-MS-based platforms to analyse the methanol and chloroform–methanol serum extracts were used. A specific metabolite extraction procedure was performed for each platform. Identified ion features in the methanol-extraction platform included fatty acids, bile acids, monoacylglycerol phospholipids, monoether glycerol phospholipids, and free sphingoid bases. The chloroform–methanol extraction platform provided coverage over glycerolipids, cholesterol esters, sphingolipids, diacylglycerophospholipids, and acyl-ether-glycerophospholipids. The lipid nomenclature and classification follow the LIPID MAPS convention (www.lipidmaps.org). Metabolite extraction procedures, chromatographic separation conditions and mass spectrometric detection conditions have been detailed previously^12^. Metabolomics data were pre-processed using the TargetLynx application manager (MassLynx 4.1; Waters Corp., Milford, MA). Intra-batch (multiple internal standard response correction) and inter-batch (variable-specific inter-batch single-point external calibration using repeat extracts of a commercial serum sample) data normalization was performed as described by Martínez-Arranz^13^.

### Statistics

Univariate analyses were performed on metabolomic-normalized data. For each metabolite, differences between subgroups were calculated as the base 2 logarithm of the fold-change. The bootstrapping method involving 1000 repetitions, each with 80% of the samples for each study group, was used to obtain a relatively unbiased estimate of fold changes and 95% confidence intervals. These values were accompanied by a significance level based on *P*-values obtained using Student’s *t* test. All calculations were performed using R statistical software package (v.3.4.1; https://www.R-project.org/).

## Results

A total of 62 PLWH were included in the study: 25 (40.3%) were receiving a darunavir-based ART (all boosted with cobicistat) and 37 (59.7%) an integrase inhibitor-based ART. The number of patients included in each group represents in both cases just over 6% of the total of patients taking that ART. The average duration of the current therapy was 5 ± 3 years in the darunavir-based group and 4 ± 3 years in the integrase inhibitor-based (*P-value* = 0.90). The main characteristics of the study population are shown in Table 1. Both groups had a similar profile for the main variables related to HIV infection. Patients in the darunavir-based group had a higher prevalence of dyslipidaemia and higher cholesterol and triglyceride concentrations than patients taking integrase inhibitor-based ART, but these differences were not significant.

**Table 1:** Baseline characteristics of study participants comparing the two study groups and the *P-value* for the comparison.

	All N = 62	DRV-based N = 25	INI-based N = 37	*P* *value*
Male (%)	51 (82.2)	23 (92.0)	28 (75.7)	0.099
Age (years)	49.3 ± 8.6	49.1 ± 7.6	49.5 ± 9.2	0.877
Current smoker (%)	38 (61.3)	15 (60.0)	23 (63.9)	0.696
BMI (Kg/m^2^)	25.3 ± 4.5	26.0 ± 5.4	24.8 ± 3.7	0.327
Hypertension (%)	12 (19.4)	6 (24.0)	6 (16.2)	0.447
Type 2 Diabetes (%)	8 (12.9)	3 (12.0)	5 (13.5)	0.862
Dyslipidemia (%)	25 (41.0)	13 (52.0)	12 (33.3)	0.145
Combined dyslipidemia (%)	12 (19.4)	6 (24.0)	6 (16.2)	0.447
Current statin treatment (%)	9 (14.5)	4 (16.0)	5 (13.5)	0.785
CV risk^§^:				0.719
Low (<5%)	24 (38.7)	9 (36.0)	15 (40.6)	
Moderate (5–10%)	25 (40.3)	11 (44.0)	14 (37.8)	
High (>10%)	13 (21.0)	5 (20.0)	8 (21.6)	
History of CV event	5 (8.1)	2 (8.0)	3 (8.1)	0.988
Total cholesterol (mg/dL)	201 ± 38	211 ± 36	194 ± 38	0.079
LDL cholesterol (mg/dL)	123 ± 35	132 ± 35	117 ± 34	0.100
HDL cholesterol (mg/dL)	51 ± 14	50 ± 15	52 ± 12	0.693
TG (mg/dL)	136 ± 73	155 ± 78	122 ± 67	0.095
ApoA-I (mg/dL)	188 ± 40	183 ± 43	193 ± 39	0.473
ApoB (mg/dL)	128 ± 31	132 ± 30	125 ± 33	0.539
Years since HIV infection	14 ± 8	15 ± 9	14 ± 8	0.570
Years on ART	12 ± 5	13 ± 6	12 ± 5	0.753
CDC-C category (%)	23 (37.1)	9 (36.0)	14 (37.8)	0.883
CD4 Count (cells/µL)	622 ± 296	608 ± 317	631 ± 286	0.770
Viral load <20 copies/mL (%)	58 (93.5)	23 (92.0)	35 (94.6)	0.683
RNA-HCV positive (%)	10 (16.1)	4 (16.0)	6 (16.2)	0.981

DRV, darunavir; INI, integrase inhibitor; BMI, body mass index; TG, triglycerides; Apo, apolipoprotein; CV, cardiovascular; CDC, Centers for Disease Control and Prevention; HCV, hepatitis C virus. ^§^Ten-year CV risk calculated with Framingham Risk Score.

The darunavir-based regimens were as follows: plus tenofovir disoproxil fumarate/emtricitabine in 10 patients, plus abacavir/lamivudine in five patients, dual therapy plus lamivudine in five patients and darunavir/cobicistat monotherapy in five patients.

The integrase inhibitor-based regimens were as follows: tenofovir alafenamide fumarate/emtricitabine/elvitegravir/cobicistat in 10 patients, tenofovir disoproxil fumarate/emtricitabine/elvitegravir/cobicistat in seven patients, abacavir/lamivudine/dolutegravir in 18 patients and tenofovir disoproxil fumarate/emtricitabine plus raltegravir in two patients.

**Figure 1:**
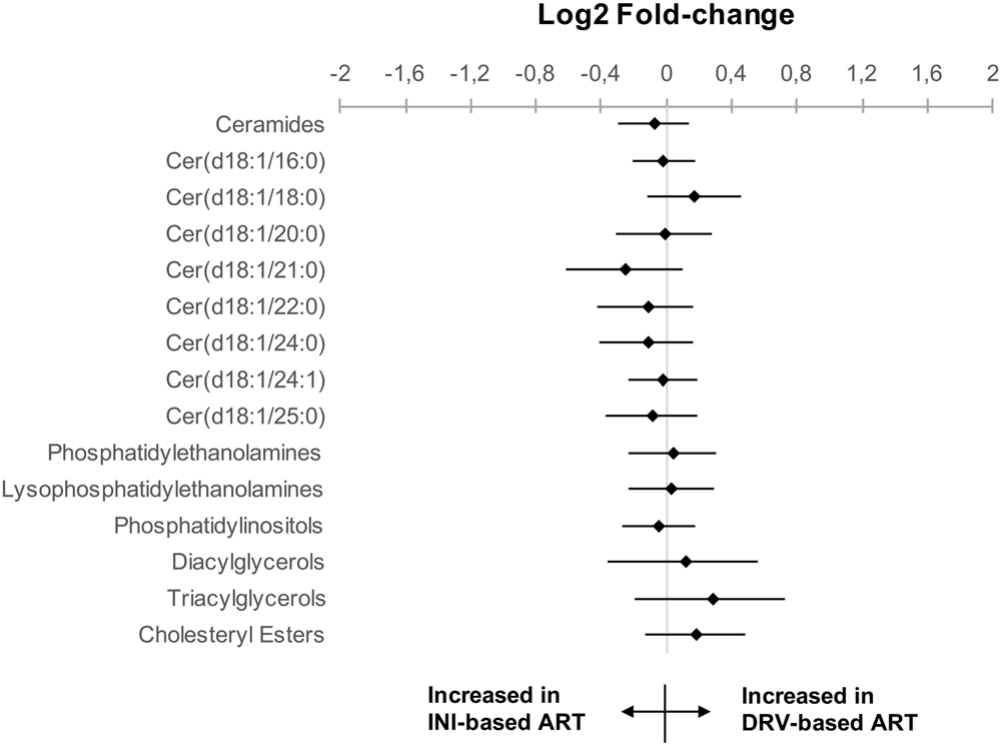
Base 2 logarithm of the fold-change (bootstrap, resampling method) and their 95% confidence interval for the comparisons between patients receiving a darunavir-based treatment and those with an integrase inhibitor-based. *P*-values obtained using Student’s *t*, in all cases *P*-value > 0.10. Cer, ceramide.

All lipid panels were compared between patients receiving darunavir-based therapy vs integrase inhibitor-based ART. The main lipid families and metabolites differed slightly between groups (log2-fold change; *P*-value): ceramides (−0.07; 0.49), phosphatidylinositols (−0.05; 0.63), diacylglycerols (0.10; 0.64), phosphatidylethanolamines (0.03; 0.78), triacylglycerols (0.27; 0.18) and lysophosphatidylethanolamines (0.03; 0.83). To compare the results visually, log2-fold changes and significance levels were used to create detailed heatmaps. Supplementary Table S1 shows the data grouped according to lipid families and Table S2 shows all metabolites. The bootstrapped log2-fold changes for the main lipid classes and ceramides involved in CV disease are depicted in Fig. 1.

The data of the main metabolites grouped according to the backbone in each of the two study groups are included and compared in Table 2. In the integrase inhibitor-based group, the use of tenofovir alafenamide fumarate significantly increases the majority of lipid fractions, when compared with tenofovir disoproxil fumarate.

**Table 2:** Mean and standard deviation of the main metabolites. Patients of the two study groups have been grouped according to the backbone. In each study group, data have been compared between different backbones using the tenofovir disoproxil fumarate/emtricitabine group as a reference.

	Darunavir-based (N = 25)			Integrase inhibitor-based (N = 37)		
	ABC/3TC N = 5	TDF/FTC N = 10	Mono/dual N = 10	ABC/3TC N = 18	TDF/FTC N = 9	TAF/FTC N = 10
Ceramides	16.33 ± 6.32	14.91 ± 3.04	15.02 ± 3.21	15.10 ± 5.29	14.68 ± 3.77	19.57 ± 4.02*
Cer(d18:1/16:0)	1.64 ± 0.39	1.32 ± 0.37	1.25 ± 0.30	1.33 ± 0.34	1.23 ± 0.21	1.35 ± 0.26
Cer(d18:1/18:0)	1.72 ± 0.64	1.47 ± 0.42	1.58 ± 0.56	1.51 ± 0.53	1.32 ± 0.33	1.65 ± 0.68
Cer(d18:1/20:0)	1.63 ± 0.59	1.29 ± 0.47	1.33 ± 0.50	1.37 ± 0.53	1.36 ± 0.29	1.97 ± 0.59*
Cer(d18:1/21:0)	1.21 ± 0.44	0.98 ± 0.24	0.95 ± 0.26	1.10 ± 0.61	1.21 ± 0.49	1.69 ± 0.54
Cer(d18:1/22:0)	1.20 ± 0.39	1.08 ± 0.31	1.11 ± 0.29	1.11 ± 0.49	1.04 ± 0.32	1.64 ± 0.57*
Cer(d18:1/24:0)	1.26 ± 0.41	1.21 ± 38	1.20 ± 0.31	1.18 ± 0.53	1.18 ± 0.36	1.74 ± 0.58*
Cer(d18:1/24:1)	1.56 ± 0.32	1.50 ± 0.42	1.49 ± 0.43	1.45 ± 0.50	1.36 ± 0.34	1.80 ± 0.35*
Cer(d18:1/25:0)	1.37 ± 0.42	1.31 ± 0.38	1.28 ± 0.42	1.37 ± 0.52	1.24 ± 0.43	1.64 ± 0.56
Phosphatidylethanolamines	37.34 ± 12.49	32.26 ± 8,84	35.10 ± 8.62	35.13 ± 10.75	29.42 ± 9.18	38.24 ± 12.5
Lysophosphatidylethanolamines	22.12 ± 9.95	19.75 ± 4.12	21.71 ± 4.76	23.88 ± 7.45*	17.02 ± 4.87	23.11 ± 5.83*
Phosphatidylinositols	5.92 ± 1.84	5.90 ± 1.76	4.88 ± 0.96	6.00 ± 1.70	4.73 ± 1.70	6.51 ± 1.72*
Diacylglycerols	7.63 ± 4.91	6.48 ± 2.12	6.39 ± 1.64	7.21 ± 4.97	5.08 ± 1.77	8.64 ± 4.13*
Triacylglycerols	198.12 ± 51.18	158.26 ± 41.50	163.19 ± 40.14	179.77 ± 44.35*	148.64 ± 47.95	204.86 ± 94.87*
Cholesteryl Esters	21.84 ± 10.12	20.76 ± 5.26	18.22 ± 3.48	17.76 ± 6.32	17.33 ± 2.40	18.98 ± 5.24

ABC/3TC, abacavir/lamivudine; TDF/FTC, tenofovir disoproxil fumarate/emtricitabine; Mono/dual, monotherapy or dual therapy (plus lamivudine); TAF/FTC, tenofovir alafenamide fumarate/emtricitabine; Cer, ceramide.

P-value < 0.05 have been represented as. *All significant P-values were >0.01.

## Discussion

In this study, we observed similar plasma lipidomic profiles in PLWH treated with darunavir/cobicistat as in those receiving an integrase inhibitor-based ART. To our knowledge, this study is the first to investigate the effects of these treatments on dyslipidaemia by analysing more than 300 metabolites.

HIV infection causes substantial inflammatory dysregulation that affects lipid metabolism and causes both qualitative and quantitative changes in several metabolites. Ceramides are associated with lipoprotein aggregation, inflammation, reactive oxygen species production and apoptosis, and seem to be an intermediate link between over-nutrition and some abnormalities related to CV risk such as insulin resistance and metabolic syndrome^10,14,15^. Few studies have analysed the influence of HIV infection and ART on these metabolic pathways in PLWH^9^, but these preliminary data show a worsening of the lipid profile with the start of ART, especially with some drugs as efavirenz, in a recently published study^16^. Ceramides are not associated with HIV infection *per se*, but the levels of some metabolites of ceramide, such as dihexosylceramide or GM3, have been found to be decreased secondarily to HIV infection, which suggests possible dysregulation of the enzyme ceramide glucosyltransferase^9^. Wong *et al*. reported that ceramides strongly predicted CV events (OR 3.44) in asymptomatic PLWH; this finding is consistent with data from studies is in HIV-uninfected people^6,7,9^. These data suggest a potential use of plasma ceramides as markers of CV risk. Measurement of ceramide level may be of particular interest in PLWH, whose CV risk scores based on traditional markers underestimate their risk.

Elevated cholesterol and triglyceride levels play an unquestionable role in CV disease. However, even after controlling these levels, there remains a residual risk that other mediators cannot change. Most of the ARTs currently used in developed countries have a minor effect on cholesterol fractions and triglycerides, but further data are needed on the entire plasma lipidomic profile to provide complete information. Darunavir/cobicistat increases cholesterol and triglyceride levels in both treatment-naive and pretreated PLWH above those observed with an integrase inhibitor-based regimen; these differences are usually statistically significant but clinically have little relevance^18^. In this study, we found no differences in the levels of ceramides or other families that can affect CV risk, such as phosphatidylethanolamines, phosphatidylinositols, cholesteryl esters or diacylglycerols. In the study by Wong *et al*., all of these lipid families were associated with future CV events in PLWH (ORs 3 to 6)^9^. Recently, an interesting study has analysed the role of ceramides in the progression of the carotid artery atherosclerosis in PLWH. Zhao *et al*. communicated a good correlation between plasma levels of C16:0 and C24:1 ceramides and immune activation and inflammation. In their data, elevated ceramide levels were associated with antiretroviral therapy use, particularly with the PIs use. We do not find differences in ceramides between the darunavir/cobicistat-based and the integrase inhibitor-based groups. Probably, in the study of Zhao, there were patients taking PIs older than darunavir/cobicistat, which are already known to have a worse lipid profile^17^.

Tenofovir disoproxil fumarate has some lipid-lowering properties, which are lost when tenofovir alafenamide fumarate is used; this has been shown by measuring fractions of cholesterol and triglycerides. In our data, despite the limitation of the sample size, patients taking tenofovir alafenamide fumarate (in all cases with an integrase inhibitor) had a worse lipid profile, when compared with tenofovir disoproxil fumarate and quantitatively worse than those who take a darunavir-based treatment, in the mail lipid metabolites. We hypothesized that, perhaps in the future, the backbone therapy is as important (or more) than the third agent, in the development of metabolic disturbances. Further studies must focus in this issue.

Lipid-lowering therapies seem to have a beneficial effect on the global lipid composition, although few studies have reported this finding^19,20^. Dietary interventions, specifically the Mediterranean diet, are effective in mitigating the deleterious effects of ceramides on CV risk, as shown by a recent report from the PREDIMED Trial^7^. In our study, few patients were taking statins (14.5%), despite the higher prevalence of dyslipidaemia (41%). This under-treatment of dyslipidaemia in these PLWH has been noted previously and seems to be explained by the low prescription rate and low adherence to lipid-lowering drugs^21^. Some investigators have hypothesized that statins are beneficial in PLWH with elevated CV risk regardless of their cholesterol levels. More studies of the levels of ceramides and other proatherogenic metabolites in PLWH will help to answer this question. The role of dietary interventions is especially interesting in PLWH and should be examined in future studies.

There are some limitations to this study. First, the small sample size limited the statistical power and did not allow us to analyse the interactions of different variables, especially the role of the different backbone therapies. Even though the groups were homogeneous, some bias was possible. This study has been designed as a proof of concept, so a sample size calculation has not been performed. The low proportion of women (<20%) limits the validity of our findings for women, and further studies are required. It was not possible to analyse specifically the role of lipid-lowering therapies in PLWH, and dietary data were not collected. The inclusion of a HIV-uninfected group as a control could potentially have helped to analyse the specific role of HIV infection in lipid disturbances. We did not include patients taking tenofovir alafenamide fumarate in the darunavir/cobicistat group, which would be desirable for a better analysis of the impact of tenofovir alafenamide fumarate on lipid disturbances.

In conclusion, we found no differences in the lipidomic profile, especially for ceramides, between patients treated with darunavir-based and those treated with an integrase inhibitor-based ART. To understand CV disease better in PLWH, especially the role of dyslipidaemia, further studies are needed to clarify the importance of multiple factors such as genetics, modifiable risk factors, diet, inflammation and senescence in addition to the role of ART exposure.

## Supplementary information

Supplementary Table S1 and S2
