# Supplementary material for: Similar plasma lipidomic profile in people living with HIV treated with a darunavir-based or an integrase inhibitor-based antiretroviral therapy
Source: Sci Rep. 2019 Nov 20;9:17184. doi: 10.1038/s41598-019-53761-7 (PMC6868233; doi:10.1038/s41598-019-53761-7)
Supplement: Supplementary file 1 — Supplementary Table S1 and S2 [file 41598_2019_53761_MOESM1_ESM.pdf]

Similar plasma lipidomic profile in people living with HIV treated with a darunavir-based or an integrase inhibitor-based antiretroviral therapy.

Authors: Alvaro Mena[1,2], Elvira Clavero[1,3], José Luis Díaz-Díaz[3], Angeles Castro[1,2].

1. Grupo de Virología Clínica, Instituto de Investigación Biomédica de A Coruña (INIBIC)-Complejo Hospitalario Universitario de A Coruña (CHUAC), Sergas. Universidad de A Coruña (UDC), Spain.
2. Unidad de Enfermedades Infecciosas, Servicio de Medicina Interna, Complejo Hospitalario Universitario de A Coruña (CHUAC), Sergas.
3. Servicio de Medicina Interna, Complejo Hospitalario Universitario de A Coruña (CHUAC), Sergas.

Table S1. The heatmap displays the log2 (fold-change) of the main lipid families included in the analysis together with the unpaired Student's t-test. For the comparisons, log transformed ion abundance ratios are depicted, as represented by the scale. Darker green and red colors indicate higher drops and elevations of the metabolite levels, respectively, in every comparison, in the darunavir (DRV)-based group using the integrase inhibitor (INI)-based group as the reference. Grey colors have been used to highlight higher significances (*P* <0.05, *P* <0.01 or *P* <0.001) in the Student´s t-test.

Class	Individual notation	DRV/C vs INI	
		**log2 (fold change)**	**Student's t-test (*P*)**
Bile Acids	*Bile Acids*	-1,401418052	**1,59E-01**
Sphingolipids	*Ceramides*	-0,072685635	**4,92E-01**
Sphingolipids	*Monohexosylceramides*	0,25171449	**1,14E-01**
Sterols	*Cholesteryl Esters*	0,177075837	**2,25E-01**
Glycerophospholipids	*Diacylglycerophosphocholine*	0,017270315	**8,66E-01**
Glycerophospholipids	*Diacylglycerophosphoethanolamine*	0,110009915	**5,49E-01**
Glycerophospholipids	*Diacylglycerophosphoinositol*	-0,052987144	**6,32E-01**
Glycerolipids	*Diacylglycerols*	0,099880799	**6,41E-01**
Fatty acids	*Fatty acids*	-0,010648056	**9,40E-01**
Fatty acids	*FA 16:1*	-0,20241088	**3,55E-01**
Fatty acids	*FA 18:1*	-0,108287132	**6,04E-01**
Fatty acids	*FA 18:2*	-0,005746891	**9,77E-01**
Fatty acids	*FA 18:3*	0,006523411	**9,77E-01**
Fatty acids	*Fatty acids omega 3*	0,25344855	**1,47E-01**
Fatty acids	*Fatty acids omega 6*	0,00345169	**9,80E-01**
Fatty acids	*Fatty acids omega 9*	-0,133597729	**5,21E-01**
Bile Acids	*Free bile acids*	-0,029716131	**9,19E-01**
Sphingolipids	*Free Sphingoid bases*	0,170053613	**1,44E-01**
Bile Acids	*Glycine-conjugated bile acids*	-2,385781012	**2,23E-01**
Glycerophospholipids	*Lysophosphatidylcholines*	0,054484732	**5,41E-01**
Glycerophospholipids	*Lysophosphatidylethanolamines*	0,02583635	**8,35E-01**
Glycerophospholipids	*Lysophosphatidylinositols*	-0,036780785	**8,01E-01**
Glycerophospholipids	*Monoacylglycerophosphocholine*	0,068656398	**4,66E-01**
Glycerophospholipids	*Monoacylglycerophosphoethanolamine*	0,055264047	**6,84E-01**
Glycerophospholipids	*Monoacylglycerophosphoinositol*	-0,036780785	**8,01E-01**
Glycerophospholipids	*1-ether, 2-acylglycerophosphocholines*	0,054797126	**5,21E-01**
Glycerophospholipids	*1-ether, 2-acylglycerophosphocholines O_plasmanyles*	0,050096032	**5,50E-01**
Glycerophospholipids	*1-ether, 2-acylglycerophosphocholines P_plasmenyles*	0,066204922	**4,85E-01**
Glycerophospholipids	*1-ether, 2-acylglycerophosphoethanolamine*	-0,007586764	**9,58E-01**
Glycerophospholipids	*1-ether, 2-acylglycerophosphoethanolamine O_plasmanyles*	-0,272954811	**9,64E-02**
Glycerophospholipids	*1-ether, 2-acylglycerophosphoethanolamine P_plasmenyles*	-0,036429564	**7,96E-01**
Glycerophospholipids	*1-Monoetherglycerophosphocholine O_plasmanyles*	-0,012312338	**9,40E-01**

Glycerophospholipids	*1-Monoetherglycerophosphocholine P_plasmenyles*	-0,151540968	**2,87E-01**
Glycerophospholipids	*1-Monoetherglycerophosphoethanolamine P_plasmenyles*	-0,244389269	**2,07E-01**
Fatty acids	*Monounsaturated fatty acids*	-0,143228647	**4,96E-01**
Glycerophospholipids	*Phosphatidylcholines*	0,032721604	**6,72E-01**
Glycerophospholipids	*PC-DHA*	0,227065979	**1,45E-01**
Glycerophospholipids	*PC 20:4*	0,023153329	**7,96E-01**
Glycerophospholipids	*PE PEMT*	0,121386387	**1,32E-01**
Glycerophospholipids	*Phosphatidylethanolamines*	0,033594995	**7,84E-01**
Glycerophospholipids	*PE-DHA*	0,165129861	**2,90E-01**
Glycerophospholipids	*PE 20:4*	-0,065413766	**6,20E-01**
Glycerophospholipids	*PC PEMT*	-0,000926719	**9,94E-01**
Glycerophospholipids	*Phosphatidylinositols*	-0,052987144	**6,32E-01**
Fatty acids	*Polyunsaturated fatty acids*	0,086588816	**4,86E-01**
Glycerolipids	*Saturated Diacylglycerols*	0,089004712	**7,01E-01**
Glycerolipids	*Saturated Triacylglycerols*	-0,12981154	**7,51E-01**
Fatty acids	*Saturated fatty acids*	-0,069163106	**6,59E-01**
Sphingolipids	*Sphingomyelins*	0,028697907	**7,34E-01**
Sterols	*Steroids*	0,339134959	**2,24E-01**
Bile Acids	*Taurine-conjugated bile acids*	-1,983679584	**2,35E-01**
Glycerolipids	*Triacylglycerols*	0,269087446	**1,81E-01**
Fatty acids	*Unsaturated fatty acids.*	0,005260837	**9,71E-01**
Glycerophospholipids	*1-Monoacylglycerophosphocholine*	0,07528425	**4,74E-01**
Glycerophospholipids	*1-Monoacylglycerophosphoethanolamine*	0,049062013	**6,11E-01**
Glycerophospholipids	*1-Monoetherglycerophosphocholine*	-0,087799407	**5,05E-01**
Glycerophospholipids	*1-Monoetherglycerophosphoethanolamine*	-0,244389269	**2,07E-01**
Glycerophospholipids	*2-Monoacylglycerophosphocholine*	0,078004485	**3,52E-01**
Glycerophospholipids	*2-Monoacylglycerophosphoethanolamine*	0,124430558	**5,78E-01**

Table S2. The heatmap displays the log2 (fold-change) of the 336 metabolites included in the analysis together with the unpaired Student's t-test. For the comparisons, log transformed ion abundance ratios are depicted, as represented by the scale. Darker green and red colors indicate higher drops and elevations of the metabolite levels, respectively, in every comparison, in the darunavir (DRV)-based group using the integrase inhibitor (INI)-based group as the reference. Grey colors have been used to highlight higher significances (*P* <0.05, *P* <0.01 or *P* <0.001) in the Student´s t-test.

**Class**	**Subclass A**	**Individual notation**	**Simplified name**	**DRV/c vs INI**	
				**log2 (fold change)**	**Student's t-test (*P*)**
Fatty acids	*Saturated fatty acids*	*12:0*	*SFA*	-0,291519373	**2,84E-01**
Fatty acids	*Saturated fatty acids*	*14:0*	*SFA*	-0,141443575	**5,95E-01**
Fatty acids	*Saturated fatty acids*	*16:0*	*SFA*	0,070721151	**6,25E-01**
Fatty acids	*Saturated fatty acids*	*17:0*	*SFA*	0,03292676	**8,50E-01**
Fatty acids	*Saturated fatty acids*	*18:0*	*SFA*	-0,043320116	**7,41E-01**
Fatty acids	*Monounsaturated fatty acids*	*16:1n-7*	*MUFA*	-0,248936142	**4,51E-01**
Fatty acids	*Monounsaturated fatty acids*	*16:1n-9*	*MUFA*	-0,269614524	**5,14E-01**
Fatty acids	*Monounsaturated fatty acids*		*MUFA*	-0,100048912	**6,33E-01**
Fatty acids	*Monounsaturated fatty acids*		*MUFA*	-0,11399557	**6,30E-01**
Fatty acids	*Monounsaturated fatty acids*	*18:1n-9*	*MUFA*	-0,108287132	**6,04E-01**
Fatty acids	*Monounsaturated fatty acids*	*20:1n-6*	*MUFA*	-0,058384187	**8,04E-01**
Fatty acids	*Polyunsaturated fatty acids*	*18:2n-6*	*PUFA*	-0,005746891	**9,77E-01**
Fatty acids	*Polyunsaturated fatty acids*	*18:3n-3*	*PUFA*	-0,07885032	**8,30E-01**
Fatty acids	*Polyunsaturated fatty acids*	*18:3n-6*	*PUFA*	0,067475387	**7,15E-01**
Fatty acids	*Polyunsaturated fatty acids*	*20:2n-6*	*PUFA*	0,002844395	**9,89E-01**
Fatty acids	*Polyunsaturated fatty acids*	*20:3n-3*	*PUFA*	0,258735663	**2,18E-02**
Fatty acids	*Polyunsaturated fatty acids*	*20:3n-9*	*PUFA*	-0,093308692	**7,15E-01**
Fatty acids	*Polyunsaturated fatty acids*		*PUFA*	-0,028692986	**8,37E-01**
Fatty acids	*Polyunsaturated fatty acids*	*20:4n-6*	*PUFA*	0,130784981	**2,13E-01**
Fatty acids	*Polyunsaturated fatty acids*	*20:5n-3*	*PUFA*	0,496733134	**1,37E-01**
Fatty acids	*Polyunsaturated fatty acids*	*22:4n-6*	*PUFA*	0,046981167	**7,54E-01**
Fatty acids	*Polyunsaturated fatty acids*	*22:5n-3*	*PUFA*	0,100133559	**6,18E-01**
Fatty acids	*Polyunsaturated fatty acids*	*22:5n-6*	*PUFA*	-0,056272783	**6,73E-01**
Fatty acids	*Polyunsaturated fatty acids*	*22:6n-3*	*PUFA*	0,32507306	**7,79E-02**
Glycerolipids	*Diacylglycerols*	*DG(32:0)*	*DG*	0,102077928	**7,24E-01**
Glycerolipids	*Diacylglycerols*	*DG(32:1)*	*DG*	0,076530051	**8,12E-01**
Glycerolipids	*Diacylglycerols*	*DG(34:0)*	*DG*	0,082365626	**6,92E-01**
Glycerolipids	*Diacylglycerols*	*DG(34:1)*	*DG*	-0,056778705	**8,05E-01**
Glycerolipids	*Diacylglycerols*	*DG(34:2)*	*DG*	0,082958206	**7,22E-01**
Glycerolipids	*Diacylglycerols*	*DG(36:2)*	*DG*	-0,265619914	**2,16E-01**
Glycerolipids	*Diacylglycerols*	*DG(36:4)*	*DG*	0,359335655	**2,27E-01**
Glycerolipids	*Diacylglycerols*	*DG(38:5)*	*DG*	0,216284794	**3,61E-01**

Glycerolipids	*Triacylglycerols*	*TG(42:0)*	*TG*	-0,496379346	**4,49E-01**
Glycerolipids	*Triacylglycerols*	*TG(44:0)*	*TG*	-0,266424962	**6,20E-01**
Glycerolipids	*Triacylglycerols*	*TG(44:1)*	*TG*	-0,123003658	**8,15E-01**
Glycerolipids	*Triacylglycerols*	*TG(44:2)*	*TG*	0,057969042	**9,01E-01**
Glycerolipids	*Triacylglycerols*	*TG(45:0)*	*TG*	-0,147457215	**7,37E-01**
Glycerolipids	*Triacylglycerols*	*TG(45:1)*	*TG*	0,047317981	**9,04E-01**
Glycerolipids	*Triacylglycerols*	*TG(46:0)*	*TG*	-0,021355205	**9,60E-01**
Glycerolipids	*Triacylglycerols*	*TG(46:1)*	*TG*	-0,113657128	**7,69E-01**
Glycerolipids	*Triacylglycerols*	*TG(46:2)*	*TG*	0,004281357	**9,91E-01**
Glycerolipids	*Triacylglycerols*	*TG(46:3)*	*TG*	0,03940612	**9,12E-01**
Glycerolipids	*Triacylglycerols*	*TG(47:0)*	*TG*	-0,078570005	**8,51E-01**
Glycerolipids	*Triacylglycerols*	*TG(47:1)*	*TG*	-0,092077738	**8,27E-01**
Glycerolipids	*Triacylglycerols*	*TG(47:2)*	*TG*	-0,002598909	**9,94E-01**
Glycerolipids	*Triacylglycerols*	*TG(48:0)*	*TG*	0,11526255	**7,52E-01**
Glycerolipids	*Triacylglycerols*	*TG(48:1)*	*TG*	-0,017449664	**9,57E-01**
Glycerolipids	*Triacylglycerols*	*TG(48:2)*	*TG*	-0,017358835	**9,53E-01**
Glycerolipids	*Triacylglycerols*	*TG(48:3)*	*TG*	0,080792112	**8,07E-01**
Glycerolipids	*Triacylglycerols*	*TG(49:0)*	*TG*	-0,059296322	**8,95E-01**
Glycerolipids	*Triacylglycerols*	*TG(49:1)*	*TG*	-0,077758582	**8,44E-01**
Glycerolipids	*Triacylglycerols*	*TG(49:2)*	*TG*	-0,03371817	**9,20E-01**
Glycerolipids	*Triacylglycerols*	*TG(49:3)*	*TG*	0,104284805	**7,28E-01**
Glycerolipids	*Triacylglycerols*	*TG(50:0)*	*TG*	0,096528102	**7,89E-01**
Glycerolipids	*Triacylglycerols*	*TG(50:1)*	*TG*	0,050940913	**8,24E-01**
Glycerolipids	*Triacylglycerols*	*TG(50:2)*	*TG*	0,021791559	**9,18E-01**
Glycerolipids	*Triacylglycerols*	*TG(50:3)*	*TG*	0,041606875	**8,32E-01**
Glycerolipids	*Triacylglycerols*	*TG(50:4)*	*TG*	0,136188811	**6,43E-01**
Glycerolipids	*Triacylglycerols*	*TG(51:1)*	*TG*	-0,10872082	**7,75E-01**
Glycerolipids	*Triacylglycerols*	*TG(51:2)*	*TG*	-0,12758125	**6,42E-01**
Glycerolipids	*Triacylglycerols*	*TG(51:3)*	*TG*	-0,021639688	**9,30E-01**
Glycerolipids	*Triacylglycerols*	*TG(51:4)*	*TG*	0,083828772	**7,25E-01**
Glycerolipids	*Triacylglycerols*	*TG(52:0)*	*TG*	-0,271681318	**5,08E-01**
Glycerolipids	*Triacylglycerols*	*TG(52:1)*	*TG*	0,036634012	**8,92E-01**
Glycerolipids	*Triacylglycerols*	*TG(52:2)*	*TG*	-0,05846636	**6,26E-01**
Glycerolipids	*Triacylglycerols*	*TG(52:3)*	*TG*	0,020009387	**8,73E-01**
Glycerolipids	*Triacylglycerols*	*TG(52:4)*	*TG*	0,093931045	**5,91E-01**
Glycerolipids	*Triacylglycerols*	*TG(52:5)*	*TG*	0,250296171	**3,54E-01**
Glycerolipids	*Triacylglycerols*	*TG(52:4)*	*TG*	0,388421249	**3,28E-01**
Glycerolipids	*Triacylglycerols*	*TG(53:0)*	*TG*	-0,128223623	**6,08E-01**
Glycerolipids	*Triacylglycerols*	*TG(53:1)*	*TG*	-0,163162978	**6,47E-01**
Glycerolipids	*Triacylglycerols*	*TG(53:2)*	*TG*	-0,253150153	**3,53E-01**
Glycerolipids	*Triacylglycerols*	*TG(53:3)*	*TG*	-0,088736149	**6,68E-01**
Glycerolipids	*Triacylglycerols*	*TG(53:4)*	*TG*	0,091347341	**6,87E-01**
Glycerolipids	*Triacylglycerols*	*TG(54:1)*	*TG*	-0,298336237	**4,38E-01**
Glycerolipids	*Triacylglycerols*	*TG(54:2)*	*TG*	-0,124666724	**5,11E-01**

Glycerolipids	*Triacylglycerols*	*TG(54:3)*	*TG*	-0,174622295	**2,21E-01**
Glycerolipids	*Triacylglycerols*	*TG(54:4)*	*TG*	-0,029940978	**8,41E-01**
Glycerolipids	*Triacylglycerols*	*TG(54:5)*	*TG*	0,051820825	**8,15E-01**
Glycerolipids	*Triacylglycerols*	*TG(54:5)*	*TG*	0,18197441	**4,85E-01**
Glycerolipids	*Triacylglycerols*	*TG(54:6)*	*TG*	0,38037885	**4,48E-01**
Glycerolipids	*Triacylglycerols*	*TG(54:6)*	*TG*	0,321092759	**2,49E-01**
Glycerolipids	*Triacylglycerols*	*TG(55:2)*	*TG*	-0,221486338	**4,35E-01**
Glycerolipids	*Triacylglycerols*	*TG(55:3)*	*TG*	-0,250598567	**3,11E-01**
Glycerolipids	*Triacylglycerols*	*TG(55:4)*	*TG*	-0,031798504	**8,76E-01**
Glycerolipids	*Triacylglycerols*	*TG(56:1)*	*TG*	-0,098879019	**8,31E-01**
Glycerolipids	*Triacylglycerols*	*TG(56:2)*	*TG*	-0,200521117	**5,46E-01**
Glycerolipids	*Triacylglycerols*	*TG(56:3)*	*TG*	-0,172702116	**3,95E-01**
Glycerolipids	*Triacylglycerols*	*TG(56:5)*	*TG*	0,103441735	**6,47E-01**
Glycerolipids	*Triacylglycerols*	*TG(56:6)*	*TG*	0,16716012	**3,51E-01**
Glycerolipids	*Triacylglycerols*	*TG(56:7)*	*TG*	0,241970408	**2,84E-01**
Glycerolipids	*Triacylglycerols*	*TG(56:7)*	*TG*	0,617895986	**5,27E-02**
Glycerolipids	*Triacylglycerols*	*TG(56:8)*	*TG*	0,807869685	**2,67E-01**
Glycerolipids	*Triacylglycerols*	*TG(56:8)*	*TG*	0,811561087	**3,79E-02**
Glycerolipids	*Triacylglycerols*	*TG(58:1)*	*TG*	0,037122772	**9,45E-01**
Glycerolipids	*Triacylglycerols*	*TG(58:2)*	*TG*	0,185039805	**7,79E-01**
Glycerolipids	*Triacylglycerols*	*TG(58:3)*	*TG*	0,019463213	**9,65E-01**
Glycerolipids	*Triacylglycerols*	*TG(58:6)*	*TG*	0,177126895	**4,06E-01**
Glycerolipids	*Triacylglycerols*	*TG(58:8)*	*TG*	0,507823027	**4,54E-02**
Glycerolipids	*Triacylglycerols*	*TG(58:9)*	*TG*	0,766414273	**7,93E-02**
Glycerolipids	*Triacylglycerols*	*TG(58:10)*	*TG*	1,422696208	**2,09E-01**
Glycerolipids	*Triacylglycerols*	*TG(60:2)*	*TG*	0,016759045	**9,78E-01**
Sterols	*Cholesteryl Ester*	*ChoE(16:0)*	*ChoE*	0,11349198	**1,82E-01**
Sterols	*Cholesteryl Ester*	*ChoE(16:1)*	*ChoE*	0,223271352	**4,05E-01**
Sterols	*Cholesteryl Ester*	*ChoE(18:0)*	*ChoE*	0,052562798	**7,01E-01**
Sterols	*Cholesteryl Ester*	*ChoE(18:1)*	*ChoE*	0,033719808	**7,54E-01**
Sterols	*Cholesteryl Ester*	*ChoE(18:2)*	*ChoE*	0,06890195	**5,31E-01**
Sterols	*Cholesteryl Ester*	*ChoE(18:3)*	*ChoE*	0,083839922	**6,65E-01**
Sterols	*Cholesteryl Ester*	*ChoE(20:3)*	*ChoE*	0,16407167	**3,94E-01**
Sterols	*Cholesteryl Ester*	*ChoE(20:4)*	*ChoE*	0,028839934	**8,14E-01**
Sterols	*Cholesteryl Ester*	*ChoE(20:5)*	*ChoE*	0,208903751	**5,30E-01**
Sterols	*Cholesteryl Ester*	*ChoE(22:4)*	*ChoE*	0,351376018	**9,43E-02**
Sterols	*Cholesteryl Ester*	*ChoE(22:5)*	*ChoE*	0,197398399	**3,87E-01**
Sterols	*Cholesteryl Ester*	*ChoE(22:6)*	*ChoE*	0,27153711	**1,30E-01**
Bile acids	*Secondary bile acids*	*Deoxycholic acid*	*BA*	0,316371118	**2,63E-01**
Bile acids	*Primary bile acids*	*Chenodeoxycholic acid*	*BA*	-0,047991859	**9,20E-01**
Bile acids		*Ursodeoxycholic + Hyodeoxycholic acid*	*BA*	-0,407737397	**3,74E-01**

Bile acids	*Glycine-conjugated bile acids*	*Glycochenodeoxycholic acid*	*BA*	-2,095723036	**9,67E-02**
Bile acids	*Glycine-conjugated bile acids*	*Glycocholic acid*	*BA*	-1,430393306	**6,25E-02**
Bile acids	*Glycine-conjugated bile acids*	*Glycodeoxycholic acid*	*BA*	-0,373705883	**3,36E-01**
Bile acids	*Glycine-conjugated bile acids*	*Glycoursodeoxycholic acid*	*BA*	-3,893584361	**3,12E-01**
Bile acids	*Taurine-conjugated bile acids*	*Taurocholic acid*	*BA*	-1,983679584	**2,35E-01**
Sterols	*Steroid sulfates*	*Dehydroepiandrosterone sulfate (DHEAS)*	*ST*	0,368442759	**2,11E-01**
Sterols	*Steroid sulfates*		*ST*	0,505615125	**5,71E-02**
Sterols	*Steroid sulfates*		*ST*	0,387310467	**3,05E-01**
Sterols	*Steroid sulfates*		*ST*	0,177487876	**4,32E-01**
Sterols	*Steroid sulfates*	*Pregnenolone sulfate*	*ST*	0,305917337	**3,40E-01**
Glycerophospholipids	*Diacylglycerophosphoethanolamine*	*PE(16:0/18:2)*	*PE_DAPE*	-0,026538798	**9,15E-01**
Glycerophospholipids	*Diacylglycerophosphoethanolamine*	*PE(16:0/20:4)*	*PE_DAPE*	-0,002582594	**9,90E-01**
Glycerophospholipids	*Diacylglycerophosphoethanolamine*	*PE(18:1/18:2)*	*PE_DAPE*	-0,30311898	**1,78E-01**
Glycerophospholipids	*Diacylglycerophosphoethanolamine*	*PE(20:5/16:0)*	*PE_DAPE*	0,668651288	**7,59E-02**
Glycerophospholipids	*Diacylglycerophosphoethanolamine*	*PE(16:0/22:6)*	*PE_DAPE*	0,298933891	**1,89E-01**
Glycerophospholipids	*Diacylglycerophosphoethanolamine*	*PE(18:0/20:4)*	*PE_DAPE*	-0,088330893	**5,90E-01**
Glycerophospholipids	*Diacylglycerophosphoethanolamine*	*PE(38:5)*	*PE_DAPE*	-0,197464672	**2,58E-01**
Glycerophospholipids	*1-ether, 2-acylglycerophosphoethanolamine*	*PE(16:1e/20:3)*	*PE_1ME2MAPE*	0,043395159	**8,42E-01**
Glycerophospholipids	*1-ether, 2-acylglycerophosphoethanolamine*	*PE(18:1e/22:6)*	*PE_1ME2MAPE*	0,061304972	**7,71E-01**
Glycerophospholipids	*1-ether, 2-acylglycerophosphoethanolamine*	*PE(18:2e/22:6)*	*PE_1ME2MAPE*	0,082521354	**6,50E-01**
Glycerophospholipids	*1-ether, 2-acylglycerophosphoethanolamine*	*PE(O-16:0/18:1)*	*PE_1ME2MAPE*	-0,272954811	**9,64E-02**
Glycerophospholipids	*1-ether, 2-acylglycerophosphoethanolamine*	*PE(P-16:0/18:2)*	*PE_1ME2MAPE*	-0,186004264	**2,31E-01**
Glycerophospholipids	*1-ether, 2-acylglycerophosphoethanolamine*	*PE(P-16:0/20:4)*	*PE_1ME2MAPE*	-0,044909896	**8,03E-01**
Glycerophospholipids	*1-ether, 2-acylglycerophosphoethanolamine*	*PE(P-18:0/18:1)*	*PE_1ME2MAPE*	-0,351061727	**2,79E-02**
Glycerophospholipids	*1-ether, 2-acylglycerophosphoethanolamine*	*PE(P-16:0/22:6)*	*PE_1ME2MAPE*	0,220710013	**1,61E-01**
Glycerophospholipids	*1-ether, 2-acylglycerophosphoethanolamine*	*PE(P-18:0/20:4)*	*PE_1ME2MAPE*	-0,069466256	**6,79E-01**
Glycerophospholipids	*1-ether, 2-acylglycerophosphoethanolamine*	*PE(P-18:1/20:4)*	*PE_1ME2MAPE*	-0,03297806	**8,58E-01**
Glycerophospholipids	*1-Monoacylglycerophosphoethanolamine*	*PE(16:0/0:0)*	*LPE_1MAPE*	0,058893341	**5,76E-01**
Glycerophospholipids	*1-Monoacylglycerophosphoethanolamine*	*PE(16:1/0:0)*	*LPE_1MAPE*	0,650528258	**1,04E-02**
Glycerophospholipids	*1-Monoacylglycerophosphoethanolamine*	*PE(18:0/0:0)*	*LPE_1MAPE*	-0,005846033	**9,55E-01**

Glycerophospholipids	*1-Monoacylglycerophosphoethanolamine*	*PE(18:1/0:0)*	*LPE_1MAPE*	-0,160713813	**3,35E-01**
Glycerophospholipids	*1-Monoacylglycerophosphoethanolamine*	*PE(18:1/0:0)*	*LPE_1MAPE*	-0,290112826	**2,81E-01**
Glycerophospholipids	*1-Monoacylglycerophosphoethanolamine*	*PE(18:2/0:0)*	*LPE_1MAPE*	-0,041893692	**7,83E-01**
Glycerophospholipids	*1-Monoacylglycerophosphoethanolamine*	*PE(18:2/0:0)*	*LPE_1MAPE*	-0,3995245	**9,42E-02**
Glycerophospholipids	*1-Monoacylglycerophosphoethanolamine*	*PE(20:3/0:0)*	*LPE_1MAPE*	-0,0691928	**6,03E-01**
Glycerophospholipids	*1-Monoacylglycerophosphoethanolamine*	*PE(20:4/0:0)*	*LPE_1MAPE*	-0,07600434	**4,47E-01**
Glycerophospholipids	*Monoacylglycerophosphoethanolamine*		*LPE_MAPE*	0,403042628	**2,13E-01**
Glycerophospholipids	*Monoacylglycerophosphoethanolamine*		*LPE_MAPE*	-0,346866318	**1,48E-01**
Glycerophospholipids	*1-Monoacylglycerophosphoethanolamine*	*PE(22:6/0:0)*	*LPE_1MAPE*	0,200865676	**9,26E-02**
Glycerophospholipids	*2-Monoacylglycerophosphoethanolamine*	*PE(0:0/16:0)*	*LPE_2MAPE*	-0,00474772	**9,73E-01**
Glycerophospholipids	*2-Monoacylglycerophosphoethanolamine*		*LPE_2MAPE*	0,621348712	**3,93E-01**
Glycerophospholipids	*2-Monoacylglycerophosphoethanolamine*	*PE(0:0/18:0)*	*LPE_2MAPE*	-0,0128505	**8,95E-01**
Glycerophospholipids	*2-Monoacylglycerophosphoethanolamine*	*PE(0:0/18:1)*	*LPE_2MAPE*	-0,220370047	**2,60E-01**
Glycerophospholipids	*2-Monoacylglycerophosphoethanolamine*	*PE(0:0/18:2)*	*LPE_2MAPE*	-0,074180351	**6,40E-01**
Glycerophospholipids	*Monoacylglycerophosphoethanolamine*		*LPE_MAPE*	-0,241803636	**1,96E-01**
Glycerophospholipids	*2-Monoacylglycerophosphoethanolamine*	*PE(0:0/20:4)*	*LPE_2MAPE*	-0,160199529	**2,55E-01**
Glycerophospholipids	*2-Monoacylglycerophosphoethanolamine*	*PE(0:0/22:6)*	*LPE_2MAPE*	0,146038697	**2,79E-01**
Glycerophospholipids	*1-Monoetherglycerophosphoethanolamine*	*PE(P-18:1/0:0)*	*LPE_1MEPE*	-0,608194999	**8,78E-02**
Glycerophospholipids	*1-Monoetherglycerophosphoethanolamine*	*PE(P-18:2/0:0)*	*LPE_1MEPE*	-0,16282193	**3,55E-01**
Glycerophospholipids	*1-Monoetherglycerophosphoethanolamine*	*PE(P-20:1/0:0)*	*LPE_1MEPE*	-0,406739331	**1,71E-01**
Glycerophospholipids	*1-Monoetherglycerophosphoethanolamine*	*PE(P-20:2/0:0)*	*LPE_1MEPE*	-0,026780541	**8,95E-01**

Glycerophospholipids	*Diacylglycerophosphocholine*	*PC(14:0/14:0)*	*PC_DAPC*	-0,478055974	**3,10E-01**
Glycerophospholipids	*Diacylglycerophosphocholine*	*PC(30:0)*	*PC_DAPC*	-0,021367515	**9,29E-01**
Glycerophospholipids	*Diacylglycerophosphocholine*	*PC(31:0)*	*PC_DAPC*	-0,111000073	**4,83E-01**
Glycerophospholipids	*Diacylglycerophosphocholine*	*PC(14:0/18:2)*	*PC_DAPC*	-0,08314425	**6,48E-01**
Glycerophospholipids	*Diacylglycerophosphocholine*	*PC(16:0/16:0)*	*PC_DAPC*	0,035144216	**7,14E-01**
Glycerophospholipids	*Diacylglycerophosphocholine*	*PC(32:1)*	*PC_DAPC*	0,058895751	**7,99E-01**
Glycerophospholipids	*Diacylglycerophosphocholine*	*PC(15:0/18:2)*	*PC_DAPC*	-0,208087701	**1,58E-01**
Glycerophospholipids	*Diacylglycerophosphocholine*	*PC(16:0/17:0)*	*PC_DAPC*	0,04325999	**8,22E-01**
Glycerophospholipids	*Diacylglycerophosphocholine*	*PC(33:1)*	*PC_DAPC*	-0,107009073	**5,13E-01**
Glycerophospholipids	*Diacylglycerophosphocholine*	*PC(14:0/20:4)*	*PC_DAPC*	-0,224171979	**2,97E-01**
Glycerophospholipids	*Diacylglycerophosphocholine*	*PC(16:0/18:0)*	*PC_DAPC*	-0,002344088	**9,80E-01**
Glycerophospholipids	*Diacylglycerophosphocholine*	*PC(16:0/18:1)*	*PC_DAPC*	-0,00690242	**9,38E-01**
Glycerophospholipids	*Diacylglycerophosphocholine*	*PC(16:0/18:2)*	*PC_DAPC*	-0,00339102	**9,55E-01**
Glycerophospholipids	*Diacylglycerophosphocholine*	*PC(16:1/18:2)*	*PC_DAPC*	-0,058975436	**7,02E-01**
Glycerophospholipids	*Diacylglycerophosphocholine*	*PC(34:3)*	*PC_DAPC*	-0,067936869	**7,43E-01**
Glycerophospholipids	*Diacylglycerophosphocholine*	*PC(15:0/20:3)*	*PC_DAPC*	-0,041388337	**8,51E-01**
Glycerophospholipids	*Diacylglycerophosphocholine*	*PC(15:0/20:4)*	*PC_DAPC*	-0,204536817	**1,84E-01**
Glycerophospholipids	*Diacylglycerophosphocholine*	*PC(16:0/19:1)*	*PC_DAPC*	-0,072297092	**5,80E-01**
Glycerophospholipids	*Diacylglycerophosphocholine*	*PC(17:0/18:1)*	*PC_DAPC*	-0,190938175	**4,07E-01**
Glycerophospholipids	*Diacylglycerophosphocholine*	*PC(17:0/18:2)*	*PC_DAPC*	-0,120995924	**2,59E-01**
Glycerophospholipids	*Diacylglycerophosphocholine*	*PC(17:1/18:1)*	*PC_DAPC*	-0,059773439	**7,26E-01**
Glycerophospholipids	*Diacylglycerophosphocholine*	*PC(17:1/18:2)*	*PC_DAPC*	-0,173517429	**2,55E-01**
Glycerophospholipids	*Diacylglycerophosphocholine*	*PC(16:0/20:4)*	*PC_DAPC*	-0,001556842	**9,88E-01**
Glycerophospholipids	*Diacylglycerophosphocholine*	*PC(16:0/20:5)*	*PC_DAPC*	0,549545383	**1,22E-01**
Glycerophospholipids	*Diacylglycerophosphocholine*	*PC(16:1/20:4)*	*PC_DAPC*	-0,066281795	**7,57E-01**
Glycerophospholipids	*Diacylglycerophosphocholine*	*PC(18:0/18:1)*	*PC_DAPC*	-0,062835316	**6,58E-01**
Glycerophospholipids	*Diacylglycerophosphocholine*	*PC(18:0/18:2)*	*PC_DAPC*	-0,053130083	**4,80E-01**
Glycerophospholipids	*Diacylglycerophosphocholine*	*PC(18:2/18:2)*	*PC_DAPC*	-0,222633728	**2,34E-01**
Glycerophospholipids	*Diacylglycerophosphocholine*	*PC(18:3/18:3)*	*PC_DAPC*	0,300172915	**1,76E-01**
Glycerophospholipids	*Diacylglycerophosphocholine*	*PC(36:3)*	*PC_DAPC*	-0,023740233	**7,85E-01**

Glycerophosp holipids	*Diacylglycerophosphocholine*	*PC(15:0/22:6)*	*PC_DAPC*	0,130164677	**5,04E-01**
Glycerophosp holipids	*Diacylglycerophosphocholine*	*PC(17:0/20:3)*	*PC_DAPC*	0,088139903	**5,54E-01**
Glycerophosp holipids	*Diacylglycerophosphocholine*	*PC(17:0/20:4)*	*PC_DAPC*	-0,177443032	**4,03E-01**
Glycerophosp holipids	*Diacylglycerophosphocholine*	*PC(37:2)*	*PC_DAPC*	-0,031583914	**8,12E-01**
Glycerophosp holipids	*Diacylglycerophosphocholine*	*PC(16:0/22:6)*	*PC_DAPC*	0,209472028	**1,10E-01**
Glycerophosp holipids	*Diacylglycerophosphocholine*	*PC(18:0/20:3)*	*PC_DAPC*	0,141618181	**2,90E-01**
Glycerophosp holipids	*Diacylglycerophosphocholine*	*PC(18:0/20:4)*	*PC_DAPC*	0,004932114	**9,68E-01**
Glycerophosp holipids	*Diacylglycerophosphocholine*	*PC(18:2/20:4)*	*PC_DAPC*	-0,035036287	**8,18E-01**
Glycerophosp holipids	*Diacylglycerophosphocholine*	*PC(20:0/18:2)*	*PC_DAPC*	-0,003817592	**9,73E-01**
Glycerophosp holipids	*Diacylglycerophosphocholine*	*PC(18:0/22:4)*	*PC_DAPC*	0,030240029	**8,57E-01**
Glycerophosp holipids	*Diacylglycerophosphocholine*	*PC(18:0/22:5)*	*PC_DAPC*	0,286874661	**5,26E-02**
Glycerophosp holipids	*Diacylglycerophosphocholine*	*PC(18:0/22:6)*	*PC_DAPC*	0,39820522	**4,79E-02**
Glycerophosp holipids	*Diacylglycerophosphocholine*	*PC(40:5)*	*PC_DAPC*	-0,065196775	**6,97E-01**
Glycerophosp holipids	*Diacylglycerophosphocholine*	*PC(40:8)*	*PC_DAPC*	0,108763975	**4,86E-01**
Glycerophosp holipids	*Diacylglycerophosphocholine*	*PC(37:5)*	*PC_DAPC*	0,087285112	**5,62E-01**
Glycerophosp holipids	*Diacylglycerophosphocholine*	*PC(38:5)*	*PC_DAPC*	-0,010006049	**9,25E-01**
Glycerophosp holipids	*Diacylglycerophosphocholine*	*PC(38:5)*	*PC_DAPC*	0,229362104	**1,74E-01**
Glycerophosp holipids	*1-ether, 2-acylglycerophosphocholine*	*PC(O-16:0/14:0)*	*PC_1ME2MAPC*	0,069178463	**5,64E-01**
Glycerophosp holipids	*1-ether, 2-acylglycerophosphocholine*	*PC(O-16:0/16:0)*	*PC_1ME2MAPC*	0,120704158	**2,79E-01**
Glycerophosp holipids	*1-ether, 2-acylglycerophosphocholine*	*PC(O-16:0/18:2)*	*PC_1ME2MAPC*	0,054000209	**7,34E-01**
Glycerophosp holipids	*1-ether, 2-acylglycerophosphocholine*	*PC(O-16:0/20:3)*	*PC_1ME2MAPC*	0,109782597	**4,87E-01**
Glycerophosp holipids	*1-ether, 2-acylglycerophosphocholine*	*PC(O-16:0/20:4)*	*PC_1ME2MAPC*	0,074663858	**5,24E-01**
Glycerophosp holipids	*1-ether, 2-acylglycerophosphocholine*	*PC(O-16:0/22:4)*	*PC_1ME2MAPC*	0,089637774	**4,20E-01**
Glycerophosp holipids	*1-ether, 2-acylglycerophosphocholine*	*PC(O-18:0/18:2)*	*PC_1ME2MAPC*	-0,019795953	**8,91E-01**
Glycerophosp holipids	*1-ether, 2-acylglycerophosphocholine*	*PC(O-18:0/20:4)*	*PC_1ME2MAPC*	0,0212637	**8,51E-01**
Glycerophosp holipids	*1-ether, 2-acylglycerophosphocholine*	*PC(O-18:0/22:4)*	*PC_1ME2MAPC*	0,000295839	**9,99E-01**
Glycerophosp holipids	*1-ether, 2-acylglycerophosphocholine*	*PC(O-18:1/18:2)*	*PC_1ME2MAPC*	-0,022437958	**8,72E-01**
Glycerophosp holipids	*1-ether, 2-acylglycerophosphocholine*	*PC(O-18:1/22:4)*	*PC_1ME2MAPC*	0,036545077	**7,25E-01**
Glycerophosp holipids	*1-ether, 2-acylglycerophosphocholine*	*PC(O-18:2/20:4)*	*PC_1ME2MAPC*	0,179968008	**2,02E-01**
Glycerophosp holipids	*1-ether, 2-acylglycerophosphocholine*	*PC(O-20:0/20:4)*	*PC_1ME2MAPC*	0,038339143	**7,76E-01**

Glycerophospholipids	*1-ether, 2-acylglycerophosphocholine*	*PC(O-22:0/20:4)*	*PC_1ME2MAPC*	0,014920961	**9,04E-01**
Glycerophospholipids	*1-ether, 2-acylglycerophosphocholine*	*PC(O-22:1/20:4)*	*PC_1ME2MAPC*	-0,006779987	**9,57E-01**
Glycerophospholipids	*1-ether, 2-acylglycerophosphocholine*	*PC(O-24:1/20:4)*	*PC_1ME2MAPC*	0,07515593	**5,40E-01**
Glycerophospholipids	*1-ether, 2-acylglycerophosphocholine*	*PC(O-34:0)*	*PC_1ME2MAPC*	0,019137654	**8,75E-01**
Glycerophospholipids	*1-ether, 2-acylglycerophosphocholine*	*PC(O-34:1)*	*PC_1ME2MAPC*	-0,09478271	**3,99E-01**
Glycerophospholipids	*1-ether, 2-acylglycerophosphocholine*	*PC(O-38:4)*	*PC_1ME2MAPC*	0,031087566	**7,78E-01**
Glycerophospholipids	*1-ether, 2-acylglycerophosphocholine*	*PC(O-38:5)*	*PC_1ME2MAPC*	0,000324935	**9,97E-01**
Glycerophospholipids	*1-ether, 2-acylglycerophosphocholine*	*PC(O-40:5)*	*PC_1ME2MAPC*	0,049608064	**6,42E-01**
Glycerophospholipids	*1-ether, 2-acylglycerophosphocholine*	*PC(O-42:6)*	*PC_1ME2MAPC*	0,198710494	**1,90E-01**
Glycerophospholipids	*1-ether, 2-acylglycerophosphocholine*	*PC(P-16:0/14:0)*	*PC_1ME2MAPC*	-0,1121971	**3,57E-01**
Glycerophospholipids	*1-ether, 2-acylglycerophosphocholine*	*PC(P-16:0/16:0)*	*PC_1ME2MAPC*	0,100468525	**2,93E-01**
Glycerophospholipids	*1-ether, 2-acylglycerophosphocholine*	*PC(P-16:0/18:1)*	*PC_1ME2MAPC*	-0,073541048	**5,44E-01**
Glycerophospholipids	*1-ether, 2-acylglycerophosphocholine*	*PC(P-16:0/18:2)*	*PC_1ME2MAPC*	-0,001648175	**9,89E-01**
Glycerophospholipids	*1-ether, 2-acylglycerophosphocholine*	*PC(P-16:0/20:4)*	*PC_1ME2MAPC*	0,114121818	**4,33E-01**
Glycerophospholipids	*1-ether, 2-acylglycerophosphocholine*	*PC(P-16:0/22:6)*	*PC_1ME2MAPC*	0,263769523	**6,03E-02**
Glycerophospholipids	*1-ether, 2-acylglycerophosphocholine*	*PC(P-17:0/20:4)*	*PC_1ME2MAPC*	-0,053707999	**7,77E-01**
Glycerophospholipids	*1-ether, 2-acylglycerophosphocholine*	*PC(P-18:0/20:4)*	*PC_1ME2MAPC*	0,208782164	**3,15E-01**
Glycerophospholipids	*1-ether, 2-acylglycerophosphocholine*	*PC(P-18:0/20:4)*	*PC_1ME2MAPC*	-0,039970126	**7,46E-01**
Glycerophospholipids	*1-ether, 2-acylglycerophosphocholine*	*PC(P-36:2)*	*PC_1ME2MAPC*	-0,140209594	**2,58E-01**
Glycerophospholipids	*1-Monoacylglycerophosphocholine*	*PC(14:0/0:0)*	*LPC_1MAPC*	-0,101888848	**5,17E-01**
Glycerophospholipids	*1-Monoacylglycerophosphocholine*	*PC(15:0/0:0)*	*LPC_1MAPC*	-0,069272054	**5,74E-01**
Glycerophospholipids	*1-Monoacylglycerophosphocholine*	*PC(16:0/0:0)*	*LPC_1MAPC*	0,045222692	**3,48E-01**
Glycerophospholipids	*1-Monoacylglycerophosphocholine*	*PC(16:1/0:0)*	*LPC_1MAPC*	-0,083545412	**5,35E-01**
Glycerophospholipids	*1-Monoacylglycerophosphocholine*	*PC(17:0/0:0)*	*LPC_1MAPC*	0,008539705	**9,33E-01**
Glycerophospholipids	*1-Monoacylglycerophosphocholine*	*PC(17:1/0:0)*	*LPC_1MAPC*	-0,110756877	**5,22E-01**
Glycerophospholipids	*1-Monoacylglycerophosphocholine*	*PC(18:0/0:0)*	*LPC_1MAPC*	0,042386666	**4,05E-01**
Glycerophospholipids	*1-Monoacylglycerophosphocholine*	*PC(18:1/0:0)*	*LPC_1MAPC*	-0,039173945	**6,47E-01**
Glycerophospholipids	*1-Monoacylglycerophosphocholine*	*PC(18:1/0:0)*	*LPC_1MAPC*	-0,028740652	**9,22E-01**
Glycerophospholipids	*1-Monoacylglycerophosphocholine*	*PC(18:2/0:0)*	*LPC_1MAPC*	0,028233529	**6,24E-01**
Glycerophospholipids	*1-Monoacylglycerophosphocholine*	*PC(18:2/0:0)*	*LPC_1MAPC*	0,06947827	**7,33E-01**

Glycerophosp holipids	*1-Monoacylglycerophosphocholine*	*PC(18:3/0:0)*	*LPC_1MAPC*	-0,094479409	**7,06E-01**
Glycerophosp holipids	*1-Monoacylglycerophosphocholine*	*PC(18:3/0:0)*	*LPC_1MAPC*	0,047963611	**8,24E-01**
Glycerophosp holipids	*1-Monoacylglycerophosphocholine*	*PC(19:0/0:0)*	*LPC_1MAPC*	-0,060831289	**7,01E-01**
Glycerophosp holipids	*1-Monoacylglycerophosphocholine*	*PC(20:0/0:0)*	*LPC_1MAPC*	-0,15929177	**2,55E-01**
Glycerophosp holipids	*1-Monoacylglycerophosphocholine*	*PC(20:1/0:0)*	*LPC_1MAPC*	-0,201858758	**1,16E-01**
Glycerophosp holipids	*1-Monoacylglycerophosphocholine*	*PC(20:1/0:0)*	*LPC_1MAPC*	-0,132091373	**5,09E-01**
Glycerophosp holipids	*1-Monoacylglycerophosphocholine*	*PC(20:2/0:0)*	*LPC_1MAPC*	0,082393084	**3,67E-01**
Glycerophosp holipids	*1-Monoacylglycerophosphocholine*	*PC(20:2/0:0)*	*LPC_1MAPC*	-0,043244696	**9,09E-01**
Glycerophosp holipids	*1-Monoacylglycerophosphocholine*	*PC(20:4/0:0)*	*LPC_1MAPC*	0,132167574	**2,95E-01**
Glycerophosp holipids	*1-Monoacylglycerophosphocholine*	*PC(20:5/0:0)*	*LPC_1MAPC*	0,487854949	**9,28E-02**
Glycerophosp holipids	*1-Monoacylglycerophosphocholine*	*PC(22:5/0:0)*	*LPC_1MAPC*	0,238955444	**6,67E-02**
Glycerophosp holipids	*1-Monoacylglycerophosphocholine*	*PC(22:5/0:0)*	*LPC_1MAPC*	0,010006339	**9,54E-01**
Glycerophosp holipids	*1-Monoacylglycerophosphocholine*	*PC(22:6/0:0)*	*LPC_1MAPC*	0,328981956	**3,70E-02**
Glycerophosp holipids	*2-Monoacylglycerophosphocholine*	*PC(0:0/14:0)*	*LPC_2MAPC*	0,035137928	**8,08E-01**
Glycerophosp holipids	*2-Monoacylglycerophosphocholine*	*PC(0:0/15:0)*	*LPC_2MAPC*	-0,119696499	**3,85E-01**
Glycerophosp holipids	*2-Monoacylglycerophosphocholine*	*PC(0:0/16:0)*	*LPC_2MAPC*	0,027531265	**6,07E-01**
Glycerophosp holipids	*2-Monoacylglycerophosphocholine*	*PC(0:0/16:1)*	*LPC_2MAPC*	-0,010478376	**9,61E-01**
Glycerophosp holipids	*2-Monoacylglycerophosphocholine*	*PC(0:0/17:0)*	*LPC_2MAPC*	-0,159614891	**3,67E-01**
Glycerophosp holipids	*2-Monoacylglycerophosphocholine*	*PC(0:0/17:1)*	*LPC_2MAPC*	-0,089202849	**6,49E-01**
Glycerophosp holipids	*2-Monoacylglycerophosphocholine*	*PC(0:0/18:0)*	*LPC_2MAPC*	-0,020224913	**8,41E-01**
Glycerophosp holipids	*2-Monoacylglycerophosphocholine*	*PC(0:0/18:1)*	*LPC_2MAPC*	-0,028255626	**8,21E-01**
Glycerophosp holipids	*2-Monoacylglycerophosphocholine*	*PC(0:0/18:2)*	*LPC_2MAPC*	-0,01140087	**8,98E-01**
Glycerophosp holipids	*2-Monoacylglycerophosphocholine*	*PC(0:0/18:3)*	*LPC_2MAPC*	-0,113765869	**6,73E-01**
Glycerophosp holipids	*2-Monoacylglycerophosphocholine*	*PC(0:0/20:0)*	*LPC_2MAPC*	-0,169977973	**2,21E-01**
Glycerophosp holipids	*2-Monoacylglycerophosphocholine*	*PC(0:0/20:1)*	*LPC_2MAPC*	-0,196914434	**7,14E-02**
Glycerophosp holipids	*2-Monoacylglycerophosphocholine*	*PC(0:0/20:2)*	*LPC_2MAPC*	0,086884673	**3,94E-01**
Glycerophosp holipids	*2-Monoacylglycerophosphocholine*	*PC(0:0/20:3)*	*LPC_2MAPC*	0,173074563	**6,76E-02**
Glycerophosp holipids	*2-Monoacylglycerophosphocholine*	*PC(0:0/20:4)*	*LPC_2MAPC*	-0,012871201	**9,20E-01**
Glycerophosp holipids	*2-Monoacylglycerophosphocholine*	*PC(0:0/20:5)*	*LPC_2MAPC*	0,449991255	**9,42E-02**
Glycerophosp holipids	*2-Monoacylglycerophosphocholine*	*PC(0:0/22:6)*	*LPC_2MAPC*	0,378905428	**3,97E-02**

Glycerophospholipids	*Monoacylglycerophosphocholine*	*LPC(19:1)*	*LPC_2MAPC*	-0,178112538	**1,76E-01**
Glycerophospholipids	*1-Monoetherglycerophosphocholine*	*PC(O-16:0/0:0)*	*LPC_1MEPC*	0,072977553	**5,27E-01**
Glycerophospholipids	*1-Monoetherglycerophosphocholine*	*PC(O-18:1/0:0)*	*LPC_1MEPC*	-0,326532949	**2,40E-01**
Glycerophospholipids	*1-Monoetherglycerophosphocholine*	*PC(O-20:0/0:0)*	*LPC_1MEPC*	0,033102366	**8,19E-01**
Glycerophospholipids	*1-Monoetherglycerophosphocholine*	*PC(O-20:1/0:0)*	*LPC_1MEPC*	-0,13128411	**4,95E-01**
Glycerophospholipids	*1-Monoetherglycerophosphocholine*	*PC(O-20:1/0:0)*	*LPC_1MEPC*	-0,225666782	**3,07E-01**
Glycerophospholipids	*1-Monoetherglycerophosphocholine*	*PC(O-20:2/0:0)*	*LPC_1MEPC*	0,032159707	**8,96E-01**
Glycerophospholipids	*1-Monoetherglycerophosphocholine*	*PC(O-22:1/0:0)*	*LPC_1MEPC*	-0,04967267	**7,78E-01**
Glycerophospholipids	*Monoetherglycerophosphocholine*	*LPC(O-22:2)*	*LPC_1MEPC*	0,126926826	**5,97E-01**
Glycerophospholipids	*1-Monoetherglycerophosphocholine*	*PC(P-16:0/0:0)*	*LPC_1MEPC*	0,000827418	**9,95E-01**
Glycerophospholipids	*1-Monoetherglycerophosphocholine*	*PC(P-18:0/0:0)*	*LPC_1MEPC*	-0,179763861	**2,38E-01**
Glycerophospholipids	*1-Monoetherglycerophosphocholine*	*PC(P-18:1/0:0)*	*LPC_1MEPC*	-0,246453653	**1,46E-01**
Glycerophospholipids	*Diacylglycerophosphoinositol*	*PI(16:0/20:4)*	*PI_DAPI*	-0,038295934	**8,25E-01**
Glycerophospholipids	*Diacylglycerophosphoinositol*	*PI(18:0/18:2)*	*PI_DAPI*	-0,08290996	**6,80E-01**
Glycerophospholipids	*Diacylglycerophosphoinositol*	*PI(18:0/20:3)*	*PI_DAPI*	-0,006929529	**9,61E-01**
Glycerophospholipids	*Diacylglycerophosphoinositol*	*PI(18:0/20:4)*	*PI_DAPI*	-0,098920665	**2,49E-01**
Glycerophospholipids	*Monoacylglycerophosphoinositol*	*LPI(16:0)*	*LPI_MAPI*	-0,00211547	**9,90E-01**
Glycerophospholipids	*Monoacylglycerophosphoinositol*	*LPI(16:0)*	*LPI_MAPI*	-0,027238041	**8,85E-01**
Glycerophospholipids	*Monoacylglycerophosphoinositol*	*LPI(18:0)*	*LPI_MAPI*	-0,108241243	**4,45E-01**
Glycerophospholipids	*Monoacylglycerophosphoinositol*	*LPI(18:0)*	*LPI_MAPI*	-0,196389114	**1,25E-01**
Glycerophospholipids	*Monoacylglycerophosphoinositol*	*LPI(18:1)*	*LPI_MAPI*	-0,247943141	**2,32E-01**
Glycerophospholipids	*Monoacylglycerophosphoinositol*	*LPI(18:1)*	*LPI_MAPI*	-0,144406154	**4,21E-01**
Glycerophospholipids	*Monoacylglycerophosphoinositol*	*LPI(18:2)*	*LPI_MAPI*	-0,079693888	**6,38E-01**
Glycerophospholipids	*Monoacylglycerophosphoinositol*	*LPI(18:2)*	*LPI_MAPI*	-0,005404628	**9,76E-01**
Glycerophospholipids	*Monoacylglycerophosphoinositol*	*LPI(20:3)*	*LPI_MAPI*	0,045360871	**7,92E-01**
Glycerophospholipids	*Monoacylglycerophosphoinositol*	*LPI(20:4)*	*LPI_MAPI*	-0,13169009	**3,42E-01**
Glycerophospholipids	*Monoacylglycerophosphoinositol*	*LPI(20:4)*	*LPI_MAPI*	-0,047433767	**6,60E-01**
Glycerophospholipids	*Monoacylglycerophosphoinositol*	*LPI(22:6)*	*LPI_MAPI*	0,216354786	**2,61E-01**
Sphingolipids	*Ceramides*	*Cer(d18:1/16:0)*	*Cer*	-0,019963275	**8,13E-01**
Sphingolipids	*Ceramides*	*Cer(d18:1/18:0)*	*Cer*	0,1643577	**2,02E-01**
Sphingolipids	*Ceramides*	*Cer(d18:1/20:0)*	*Cer*	-0,015050874	**9,14E-01**

Sphingolipids	*Ceramides*	*Cer(d18:1/21:0)*	*Cer*	-0,26578587	**1,31E-01**
Sphingolipids	*Ceramides*	*Cer(d18:1/22:0)*	*Cer*	-0,112458542	**4,42E-01**
Sphingolipids	*Ceramides*	*Cer(40:2)*	*Cer*	-0,200732353	**1,26E-01**
Sphingolipids	*Ceramides*	*Cer(d18:1/23:0)*	*Cer*	-0,219888438	**7,28E-02**
Sphingolipids	*Ceramides*	*Cer(d18:1/24:0)*	*Cer*	-0,121382418	**3,99E-01**
Sphingolipids	*Ceramides*	*Cer(d18:1/24:1)*	*Cer*	-0,026739391	**8,00E-01**
Sphingolipids	*Ceramides*	*Cer(42:3)*	*Cer*	-0,097647479	**4,03E-01**
Sphingolipids	*Ceramides*	*Cer(d43:1)*	*Cer*	0,001498324	**9,94E-01**
Sphingolipids	*Ceramides*	*Cer(d18:1/25:0)*	*Cer*	-0,092600083	**4,75E-01**
Sphingolipids	*Sphingomyelin*	*SM(43:2)*	*SM*	0,207278213	**2,18E-01**
Sphingolipids	*Sphingomyelin*	*SM(31:1)*	*SM*	-0,249362408	**1,35E-01**
Sphingolipids	*Sphingomyelin*	*SM(38:0)*	*SM*	0,240964534	**1,95E-01**
Sphingolipids	*Sphingomyelin*	*SM(42:1)*	*SM*	0,107040586	**4,14E-01**
Sphingolipids	*Sphingomyelin*	*SM(43:1)*	*SM*	0,050481189	**8,09E-01**
Sphingolipids	*Sphingomyelin*	*SM(32:1)*	*SM*	-0,090035824	**4,39E-01**
Sphingolipids	*Sphingomyelin*	*SM(33:1)*	*SM*	-0,17429588	**7,66E-02**
Sphingolipids	*Sphingomyelin*	*SM(d18:0/14:0)*	*SM*	-0,002585855	**9,84E-01**
Sphingolipids	*Sphingomyelin*	*SM(d18:0/16:0)*	*SM*	0,084209878	**4,06E-01**
Sphingolipids	*Sphingomyelin*	*SM(d18:0/18:0)*	*SM*	0,353139314	**2,10E-01**
Sphingolipids	*Sphingomyelin*	*SM(d18:0/22:0)*	*SM*	0,178517839	**4,21E-01**
Sphingolipids	*Sphingomyelin*	*SM(d18:1/12:0)*	*SM*	-0,325206294	**6,43E-02**
Sphingolipids	*Sphingomyelin*	*SM(d18:1/16:0)*	*SM*	0,028372622	**6,52E-01**
Sphingolipids	*Sphingomyelin*	*SM(d18:1/17:0)*	*SM*	0,012552703	**8,81E-01**
Sphingolipids	*Sphingomyelin*	*SM(d18:1/18:0)*	*SM*	0,190040585	**8,64E-02**
Sphingolipids	*Sphingomyelin*	*SM(36:2)*	*SM*	0,114016588	**2,31E-01**
Sphingolipids	*Sphingomyelin*	*SM(38:1)*	*SM*	0,032603038	**7,63E-01**
Sphingolipids	*Sphingomyelin*	*SM(39:1)*	*SM*	-0,111383672	**4,33E-01**
Sphingolipids	*Sphingomyelin*	*SM(d18:1/22:0)*	*SM*	0,062070637	**5,78E-01**
Sphingolipids	*Sphingomyelin*	*SM(d18:1/23:0)*	*SM*	0,014474906	**9,02E-01**
Sphingolipids	*Sphingomyelin*	*SM(d18:1/23:1)*	*SM*	-0,018377193	**8,75E-01**
Sphingolipids	*Sphingomyelin*	*SM(42:3)*	*SM*	0,107769208	**1,68E-01**
Sphingolipids	*Sphingomyelin*	*SM(d18:1/24:1)*	*SM*	0,14446911	**6,27E-02**
Sphingolipids	*Sphingomyelin*	*SM(d18:1/25:0)*	*SM*	0,178010705	**1,23E-01**
Sphingolipids	*Sphingomyelin*	*SM(d18:2/14:0)*	*SM*	-0,132510196	**3,48E-01**
Sphingolipids	*Sphingomyelin*	*SM(d18:2/16:0)*	*SM*	-0,015354426	**8,72E-01**
Sphingolipids	*Sphingomyelin*	*SM(d18:2/20:0)*	*SM*	-0,008917748	**9,30E-01**
Sphingolipids	*Sphingomyelin*	*SM(d18:2/22:0)*	*SM*	-0,008464985	**9,32E-01**
Sphingolipids	*Sphingomyelin*	*SM(d18:2/23:0)*	*SM*	-0,099080738	**4,39E-01**
Sphingolipids	*Monohexosylceramides*	*CMH(d18:1/24:0)*	*CMH*	0,301796981	**5,84E-02**
Sphingolipids	*Monohexosylceramides*	*CMH(d18:1/23:0)*	*CMH*	0,199431513	**2,43E-01**
Sphingolipids	*Free Sphingoid base*	*Sphingosine-1-phosphate*	*FSB*	0,170053613	**1,44E-01**
